# A signature based on five immune-related genes to predict the survival and immune characteristics of neuroblastoma

**DOI:** 10.1186/s12920-022-01400-y

**Published:** 2022-11-23

**Authors:** KeXin Ma, PeiPei Zhang, Yu Xia, Lin Dong, Ying Li, Liu Liu, YaJuan Liu, YouJun Wang

**Affiliations:** 1 Department of Pediatrics, The Fifth Affiliated Hospital of Zhengzhou University, No. 3 Kangfuqian Street, Zhengzhou, China; 2 Department of Neonatology, Children’s Hospital, Capital Institute of Pediatrics, Beijing, China

**Keywords:** Neuroblastoma, MYCN amplification, Immune-related gene, Prognosis

## Abstract

**Background:**

MYCN amplification (MNA) has been proved to be related to poor prognosis in neuroblastoma (NBL), but the MYCN-related immune signatures and genes remain unclear.

**Methods:**

Enrichment analysis was used to identify the significant enrichment pathways of differentially expressed immune-related genes (DEIRGs). Weight gene coexpression network analysis (WGCNA) was applied to reveal the correlation between these DEIRGs and MYCN status. Univariate and multivariate Cox analyses were used to construct risk model. The relevant fractions of immune cells were evaluated by CIBERSORT and single-sample gene set enrichment analysis (ssGSEA).

**Results:**

Five genes, including CHGA, PTGER1, SHC3, PLXNC1, and TRIM55 were enrolled into the risk model. Kaplan–Meier survival analysis and receiver operating characteristic (ROC) curve showed that our model performed well in predicting the outcomes of NBL (3-years AUC = 0.720, 5-year AUC = 0.775, 10-years AUC = 0.782), which has been validated in the GSE49711 dataset and the E-MTAB-8248 dataset. By comparing with the tumor immune dysfunction and exclusion (TIDE) and tumor inflammation signature (TIS), we further proved that our model is reliable. Univariate and multivariate Cox regression analyses indicated that the risk score, age, and MYCN can serve as independent prognostic factors in the E-MATB-8248. Functional enrichment analysis showed the DEIRGs were enriched in leukocyte adhesion-related signaling pathways. Gene set enrichment analysis (GSEA) revealed the significantly enriched pathways of the five MYCN-related DEIRGs. The risk score was negatively correlated with the immune checkpoint CD274 (PD-L1) but no significant difference with the TMB. We also confirmed the prognostic value of our model in predicting immunotherapeutics.

**Conclusion:**

We constructed and verified a signature based on DEIRG that related to MNA and predicted the survival of NBL based on relevant immune signatures. These findings could provide help for predicting prognosis and developing immunotherapy in NBL.

**Supplementary Information:**

The online version contains supplementary material available at 10.1186/s12920-022-01400-y.

## Introduction

Neuroblastoma (NBL) is the most common embryonal solid tumor of infancy, which accounts for 7–8% of malignancies and 15% of cancer-related mortality in children [1]. It is a heterogeneous tumor deriving from neural crest cells (NCCs) [2]. The genetic, morphological, and clinical heterogeneity have been described at multiple levels, including anatomical localization, histology, genomics/molecular profile, and cellular and molecular levels. The considerable heterogeneity contributes to the clinical and prognostic diversity. Therefore, children with the same stage usually have different outcomes. Although treatments of NBL, for example, high-dose myeloablative chemotherapy, molecular-targeted therapy, autologous hematopoietic stem cell transplantation (AHSCT), and immunotherapy have significantly prolonged the survival of patients [3], NBL is still a life-threatening malignancy with 5-year survival rate less than 50% [4]. Therefore, it is urgently required to develop and validate novel prognostic predictors to guide the treatment of NBL.

MYCN amplification (MNA) accounts for about 20–25% of all primary tumors [5]. which is an initiating event that drives the development of high-risk NBL and is strongly associated with high-risk disease and poor prognosis [6]. MYCN affects not only gene expression but also epigenetic factors [7]. It has been used as a biomarker of risk stratification, but the specific related immune features and genes remain unclear. Therefore, it is necessary to establish computational models of the immune-related genes in different MYCN status groups.

In recent years, treatments based on the tumor microenvironment (TME) have attracted attention. TME plays a significant role in tumor progression by providing a growth environment, reducing the efficacy of anti-tumor drugs, and helping tumor cells evade immune surveillance. Overall, tumor-infiltrating immune cells (TIICs) and immune-related genes (IRGs) are essential components of the TME [8, 9]. Studies have shown that the stromal and immune signatures are related to the survival of NBL. However, the specific mechanisms of TME related to tumor progression remain unclear. Therefore, we tried to analyze the relationship between the genes and TME alternations.

In this study, we developed and validated individualized prognostic characteristics of NBL based on the MYCN-related DEIRGs, and then compared the immune components between the two score groups. These results may provide insights for further prediction of the prognosis of NBL patients.

## Material and methods

### Public datasets

We downloaded the mRNA matrix, somatic mutation, and clinical data from the Therapeutically Applicable Research to Generate Effective Treatments (TARGET) database (www.ocg.cancer.gov/programs/target).

The validation cohorts were downloaded from the Gene Expression Omnibus (GEO) database (GSE49711, n = 498, www.ncbi.nlm.nih.gov/geo/) and the ArrayExpress database (E-MTAB-8248, n = 223, www.ebi.ac.uk/arrayexpress). The immunotherapy dataset (IMvigor210, n = 298) was downloaded from the “IMvigor210CoreBiologies” R package (http://research-pub.gene.com/IMvigor210CoreBiologies/packageVersions). 298 patients with metastatic urothelial cancer in the IMvigor210 dataset treated with an anti-PD-L1 agent (atezolizumab). IRGs list was obtained from the ImmPort database (www.immport.org/home) and the InnateDB database (www.innatedb.ca).

### Analysis of DEIRGs and gene enrichment analysis

According to the MYCN status (MNA or non-MNA), we identified the differentially expressed genes (DEGs) with a false discovery rate (FDR) < 0.05 and |logFC|> 1 between the two groups in the TARGET cohort using "limma" R package. Then we extracted differentially expressed immune-related genes (DEIRGs) from DEGs based on the IRGs list. These DEGs and DEIRGs were shown in the heatmap. In order to gain new insights into the mechanism and pathways related to these DEIRGs, we performed Gene Ontology (GO) and Kyoto Encyclopedia of Genes and Genomes (KEGG) [10–12] enrichment analyses.

### WGCNA to identify key modules and hub genes

To screen hub genes associated with MYCN status, weight gene coexpression network analysis (WCGNA) was carried out based on the expression of these DEIRGs by “WGCNA” R package. We first calculated the degree of adjacency between every two genes and applied the standard scale-free network to evaluate the optimal soft threshold power. Topological overlap matrix (TOM) was used to reduce the effects of noise and spurious associations. TOM-based dissimilarity was used to form modules through dynamic tree cutting. The clustering dendrogram of genes was created through a dendrogram with colored assignments when cut height = 0.2, minModuleSize = 25. To select key modules associated with MNA, *p* < 0.05 was considered significant. Network screening was used to visualize hub genes in the key modules with Cytoscape 3.5.1 software.

### Construction of a prognostic signature based on MYCN-related DEIRGs

Univariate Cox regression analysis was used to select prognostic genes located in the key modules. Multivariate Cox regression analysis was used to calculate the regression coefficients for each gene. We constructed a prognostic risk model based on these genes. The formula was as follows: risk score = β1 * X1 + β2 * X2 + … + βn* Xn (β, risk coefficient; X, the expression of a specific gene). Patients were assigned to the high- or low-risk group based on the median risk score. Next, we performed univariate and multivariate analyses to identify whether the risk score can serve as an independent factor in NBL. The tumor immune dysfunction and exclusion (TIDE) and tumor inflammation signature (TIS) have been proved to be effective. By comparing with TIDE and TIS, we further proved that our model is reliable. Moreover, we evaluated the correlations between the risk model and clinical factors, including age (age <  = 1.5, age > 1.5), gender (female, male), INSS stage (stage 2, 3, 4), MYCN status (amplification, unamplification). We also calculated the proportions of patients in different age groups.

### Gene set enrichment analysis

Gene set enrichment analysis (GSEA) was used to identify significantly enriched pathways in the two groups. The “c2.cp.kegg.v7.4.symbols.gmt” gene set collection in the MSigDB database was chosen as the reference. *p* < 0.05 was considered statistically significant.

### Analysis of tumor mutation burden

We downloaded the somatic mutation data of 209 patients from the TARGET database and extracted relevant information through Perl script (www.perl.org/), then we estimated the tumor mutation burden (TMB) values. TMB = (total count of variants) / (the whole length of exons). The R package “maftools” was used to display specific mutation information for the top 20 genes in different groups.

### The correlation between 5-DEIRGs and tumor-infiltrating immune cells

The R package “CIBERSORT” was used to quantify 22 kinds of human immune cell phenotypes in each sample and infer their relative proportions. Gene set LM22 was used as the reference. In each sample, the sum of the proportions of 22 immune cells was equal to 1. We set permutations = 1000 to improve the accuracy. Only samples with a CIBERSORT *p* < 0.05 were selected for the following analysis. TIICs infiltration was divided in low- or high- abundance. We also compared the expression difference of immune checkpoint gene CD274 (PD-L1) in two risk groups and the prognostic value of our model in predicting immunotherapeutics.

### Single-sample gene set enrichment analysis

We performed single-sample gene set enrichment analysis (ssGSEA) to evaluate the activities of 13 immune-related functions enrichment pathways and infiltration scores of 16 immune cells of each example using the R package "GSVA".

### Statistical analysis

Survival analysis was carried out by the K-M analysis, and the log-rank test was applied between groups. Univariate and multivariate Cox proportional-hazards models were utilized for model construction. The receiver operating characteristic (ROC) curve was used to estimate the predictive ability of our model. Wilcoxon test was used to analyze the correlation between the risk model and clinical features and immune characteristics. For all tests, *p* < 0.05 was considered to be significant. Data analysis in our study was performed by R language version 4.0.5 (www.r-project.org/) with the following packages: “glment,” “survminer,” “survival ROC,” and “clusterProfile.”

## Results

### Identification of DEIRG

**Fig. 1:**
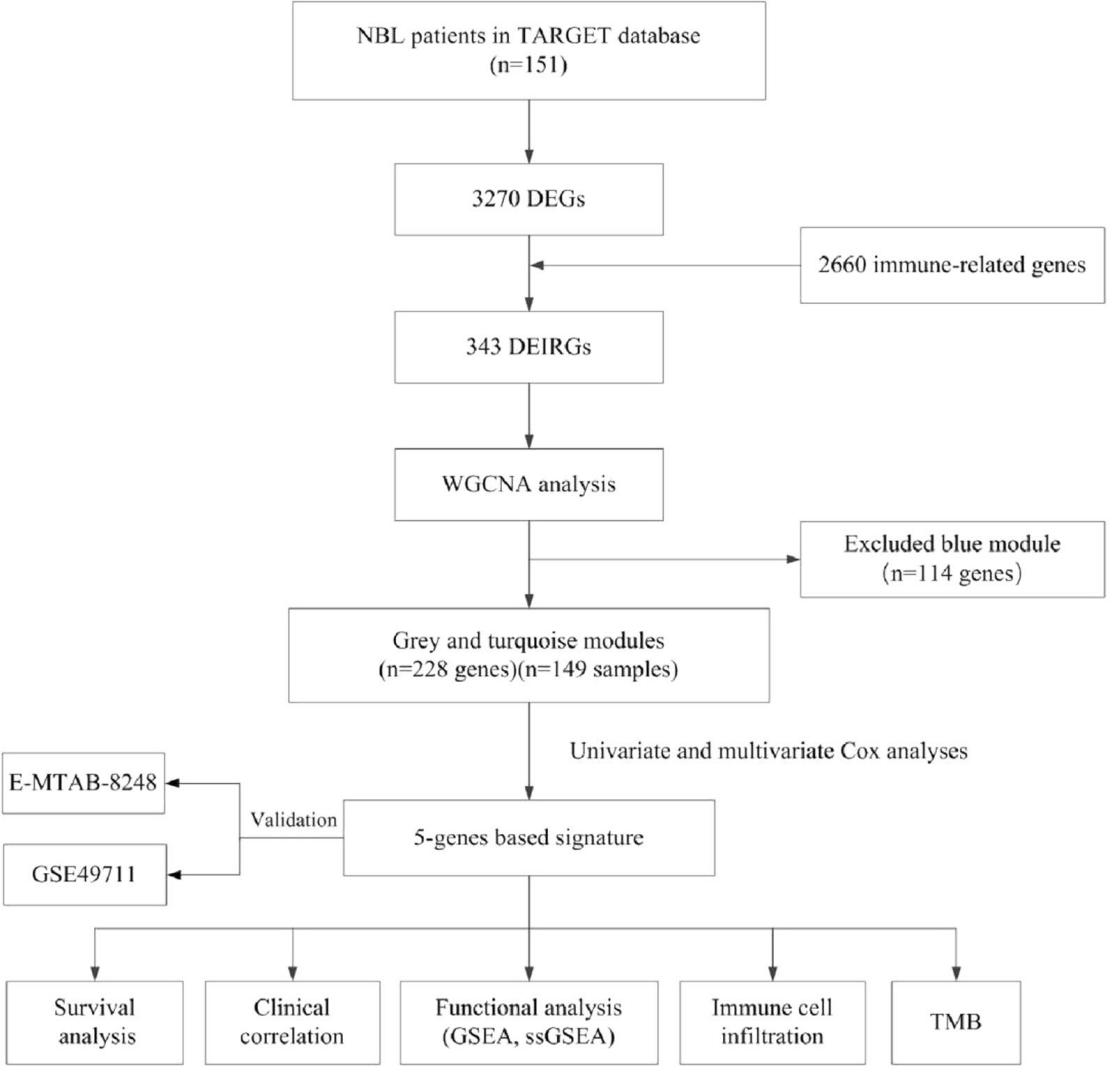
The flow chart of this study

**Fig. 2:**
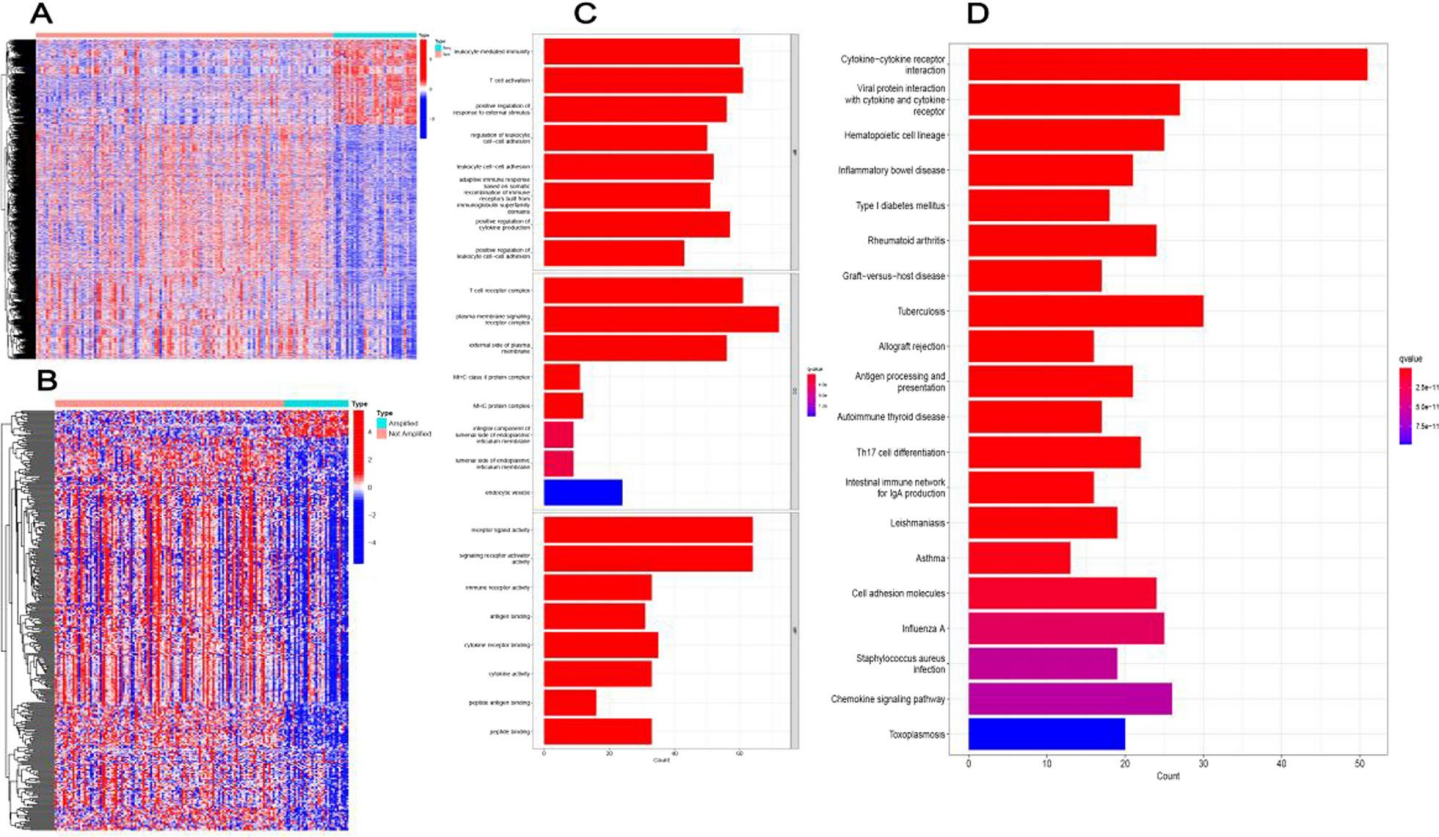
Identification of DEIRGs and enrichment analysis in the TARGET cohort. Heatmap of the (**A**) DEGs and **B** DEIRGs in the MNA group. **C** GO, and **D** KEGG enrichment analysis of the 343 DEIRGs. *p*- and q-value < 0.05 were considered significantly

We downloaded transcriptome profiling and clinical databases of 151 NBL patients from the pediatric TARGET database. Transcriptome profiling of 498 NBL patients were downloaded from the GEO database and transcriptome profiling of 223 NBL patients were downloaded from the ArrayExpress, which served as the external validation groups. The corresponding flow chart is shown in Fig. 1A. We analyzed the DEGs between the MNA group (n = 33) and the non-MNA group (n = 118). A total of 3270 DEGs were identified, which were displayed in the heatmap (Fig. 2A). From ImmPort and InnateDB databases, we obtained 2660 immune-related genes (IRGs). We extracted 343 DEIRGs from the 3270 DEGs based on the IRGs list, among which 320 genes were down‐regulated and 23 genes were up‐regulated in the MNA group compared with non-MNA group (Fig. 2B).

Function enrichment analyses were performed using KEGG and GO analyses. By GO analysis (Fig. 2C), DEIRGs were significantly enriched in leukocyte-mediated immunity, T cell activation, signaling receptor activator activity, receptor-ligand activity, T cell receptor complex, and plasma membrane signaling receptor complex. KEGG analysis showed that these DEIRGs were mainly enriched in cytokine-cytokine receptor interaction, tuberculosis, and chemokine signaling pathway (Fig. 2D). In brief, these DEIRGs play immune roles in NBL mainly involved in transmitting various signaling pathways.

### WGCNA to identify key modules and hub genes

**Fig. 3:**
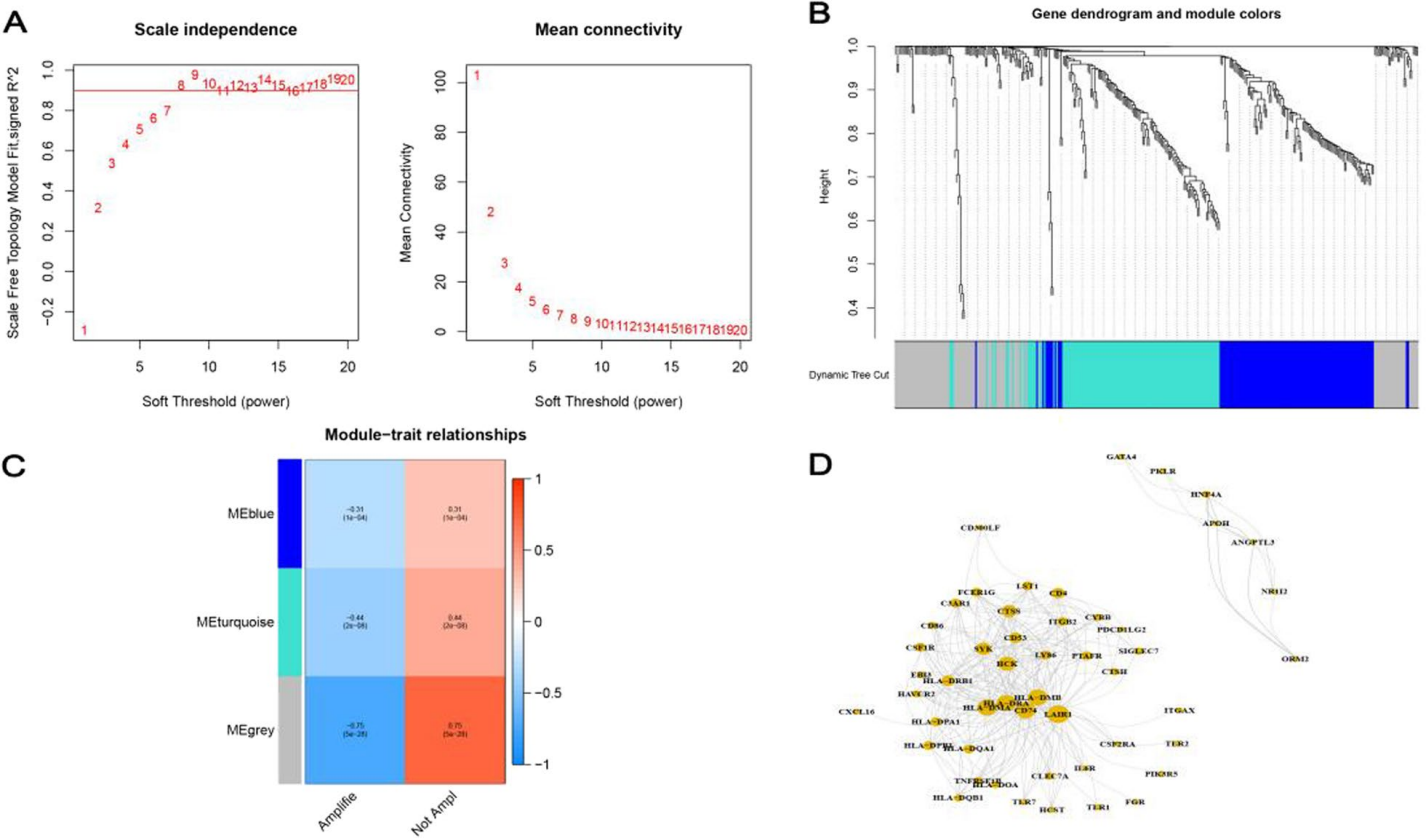
WGCNA screening key modules and hub genes related to MNA. **A**, **B** The scale-free network and clustering tree of co-expression modules. **C** Grey, turquoise and blue co-expression modules were identified correlated with MNA. **D** visualization of the grey and turquoise network modules

To further identify DEIRGs related to MYCN status, we performed WGCNA to reveal the correlation between 343 DEIRGs and MYCN status. The scale-free network was constructed based on the expression profile of 343 DEIRGs with a soft threshold power of 8 (Fig. 3A). Next, a total of 3 modules were identified by the dynamic cutting tree (minModuleSize = 25, cut height = 0.2) (Fig. 3B) and results showed that the turquoise (R = 0.44, *p* < 0.001) and grey (R = 0.75, *p* < 0.001) modules were closely correlated with MNA (Fig. 3C). 228 DEIRGs (n = 149 samples) in the grey and turquoise modules were used to identify hub genes using the Cytoscape graph. The hub genes were located in the center of the modules: the larger the circle, the more likely it to be a core gene (Fig. 3D).

### Construction and validation of the prognostic signature

**Fig. 4:**
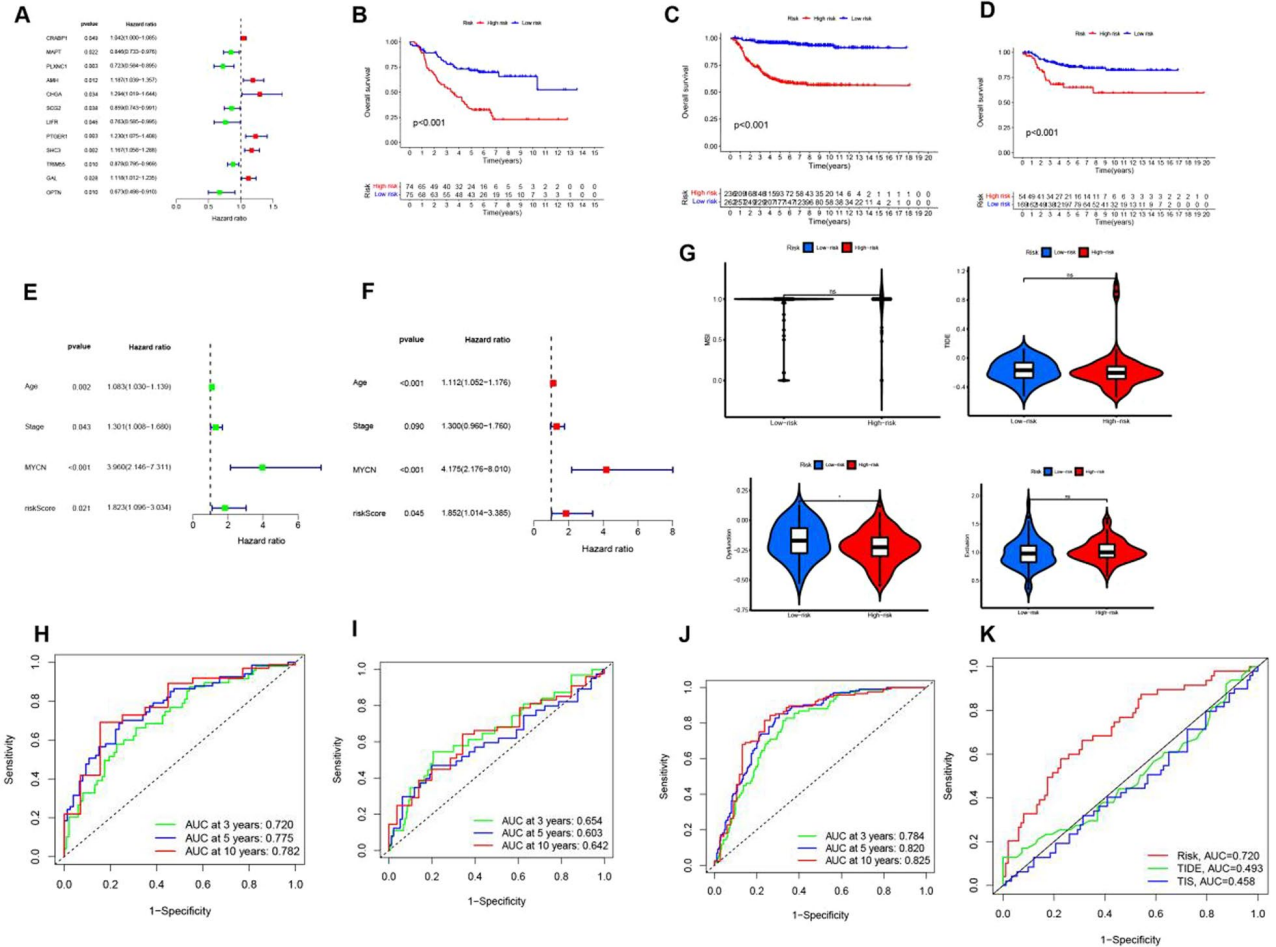
Identification of prognostic genes, construction and validation of the prognostic model. **A** The forest plot showed the relationship between the gene expression and OS. Kaplan–Meier survival analysis of patients in the high or low-risk group in the **B** TARGET, **C** GSE49711, and **D** E-MTAB-8248 datasets. The independent prognostic factors in the E-MTAB-8248 dataset was assessed by **E** univariate and **F** multivariate Cox analysis. **G** TIDE, dysfunction, MSI and exclusion score in the high and low-risk groups. **H**–**J** ROC curves for predicting survival at 3, 5, and 10 years of NBL patients in our model, the E-MTAB-8248, and the GSE49711 datasets, respectively. **K** Multivariable ROC curve compared with the TIDE and TIS risk models. *p*-value < 0.05 was considered significant

A total of 228 DEIRGs (n = 149 samples) originated from grey and turquoise modules that related to MNA were used for further analysis. As shown in the univariate Cox regression analysis, we screened 12 prognostic genes, including CRABP1, AMH, CHGA, PTGER1, SHC3, GAL, MAPT, PLXNC1, SCG2, LIFR, TRIM55, and OPTN (Fig. 4A). We applied the multivariate stepwise method (both forward and backward) to screen optimal genes to construct the prognostic model. Finally, five genes, including CHGA, PTGER1, SHC3, PLXNC1, and TRIM55 were enrolled and the risk score of each patient was calculated based on the following formula: The risk score = (-0.326 * the expression of CHGA) + (0.384 * the expression of PTGER1) + (0.199 * the expression of SHC3) + (0.114 * the expression of PLXNC1) + (-0.114 * the expression of TRIM55). Patients were assigned to the low- or high-risk group based on the median value. As shown in the K-M curves, the high-level expression of PLXNC1 and TRIM55 showed better survival outcomes in the high-risk group, while the high-level expression of CHGA, SHC3, and PTGER1 showed poorer survival outcomes in the high-risk group (Additional file 1: Fig. S1A–E). K–M analysis showed that the low-risk group patients had significantly better survival prognosis (*P* < 0.001) (Fig. 4B), which was validated in the GSE49711 dataset (*P* < 0.001) (Fig. 4C) and the E-MTAB-8248 dataset (*P* < 0.001) (Fig. 4D). In addition, the univariate and multivariate Cox regression analyses indicated that the risk score served as an independent prognostic factor in NBL patients in our model (Additional file 1: Fig. S2A, B). Subsequently, we identified independent prognostic factors in the E-MTAB-8248 and GSE49711 datasets. In the E-MTAB-8248 dataset, the univariate Cox regression analysis indicated that the age, stage, MYCN, and risk score were associated with the patient’s prognosis (Fig. 4E). Multivariate Cox regression analysis indicated that the age, MYCN, and risk score were associated with the prognosis (Fig. 4F). The independent prognostic factors in the GSE49711 dataset are shown in the Additional file 1:Fig. S3A, B. Accordingly, the risk score, age, and MYCN all can serve as independent prognostic factors for NBL patients.

TIDE was used to compare the immunotherapy response between the two groups, and the higher the TIDE prediction score, the higher the probability of immune evasion. The low-risk group patients had a higher dysfunction (*p < 0.05), while the TIDE, MSI, and exclusion showed no significant differences in the two risk groups (Fig. 4G). Our model performed well in predicting the outcomes of NBL (3-years AUC = 0.720, 5-year AUC = 0.775, 10-years AUC = 0.782) (Fig. 4H), which has been validated in the GSE49711 dataset (3-years AUC = 0.784, 5-year AUC = 0.820, 10-years AUC = 0.825) (Fig. 4J) and the E-MTAB-8248 dataset (3-years AUC = 0.654, 5-year AUC = 0.603, 10-years AUC = 0.642) (Fig. 4I). The AUC values of risk model, TIDE, and TIS was 0.720, 0.493, and 0.458, respectively (Fig. 4K). Overall, the 5-MYCN-related DEIRGs signature has a better predictive ability.

### Comparison of clinical features with the risk model

**Fig. 5:**
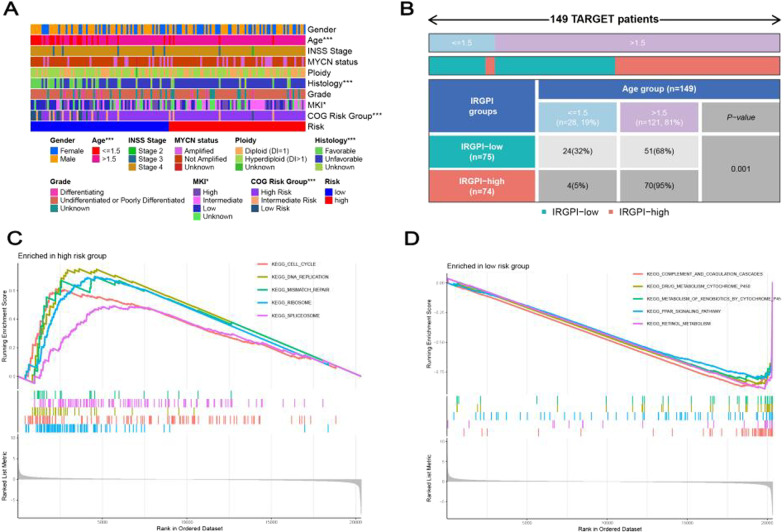
The relationship between the risk model and clinical characteristics. **A** Heatmap of correlation between the risk model and clinical features in TARGET cohort, **B** chi-square test ratio. *p < 0.05; **p < 0.01; ***p < 0.001. Pathways enriched in NBL patients in the **C** high-risk and **D** low-risk groups

Age, histology, COG (***P < 0.001), and MKI (*P < 0.05) showed differences in the two risk groups (Fig. 5A). We calculated the proportions of patients in two age groups (age ≤ 1. 5, age > 1.5), and the results showed in Fig. 5B. All in all, some known clinical features of the prognosis [14] were associated with our constructed the 5-gene risk model.

### Gene set enrichment analysis

We performed GSEA to explore the possible signaling pathways and mechanisms. The results showed that Cell cycle, mismatch repair, DNA replication, ribosome, and spliceosome pathway were highly enriched in the high-risk group (Fig. 5C). Complement and coagulation cascades, metabolism of xenobiotics by cytochrome p450, PPAR signaling pathway, drug metabolism cytochrome p450, and retinol metabolism were highly enriched in the low-risk group (Fig. 5D).

### Correlation of tumor microenvironment with patients’ prognosis

**Fig. 6:**
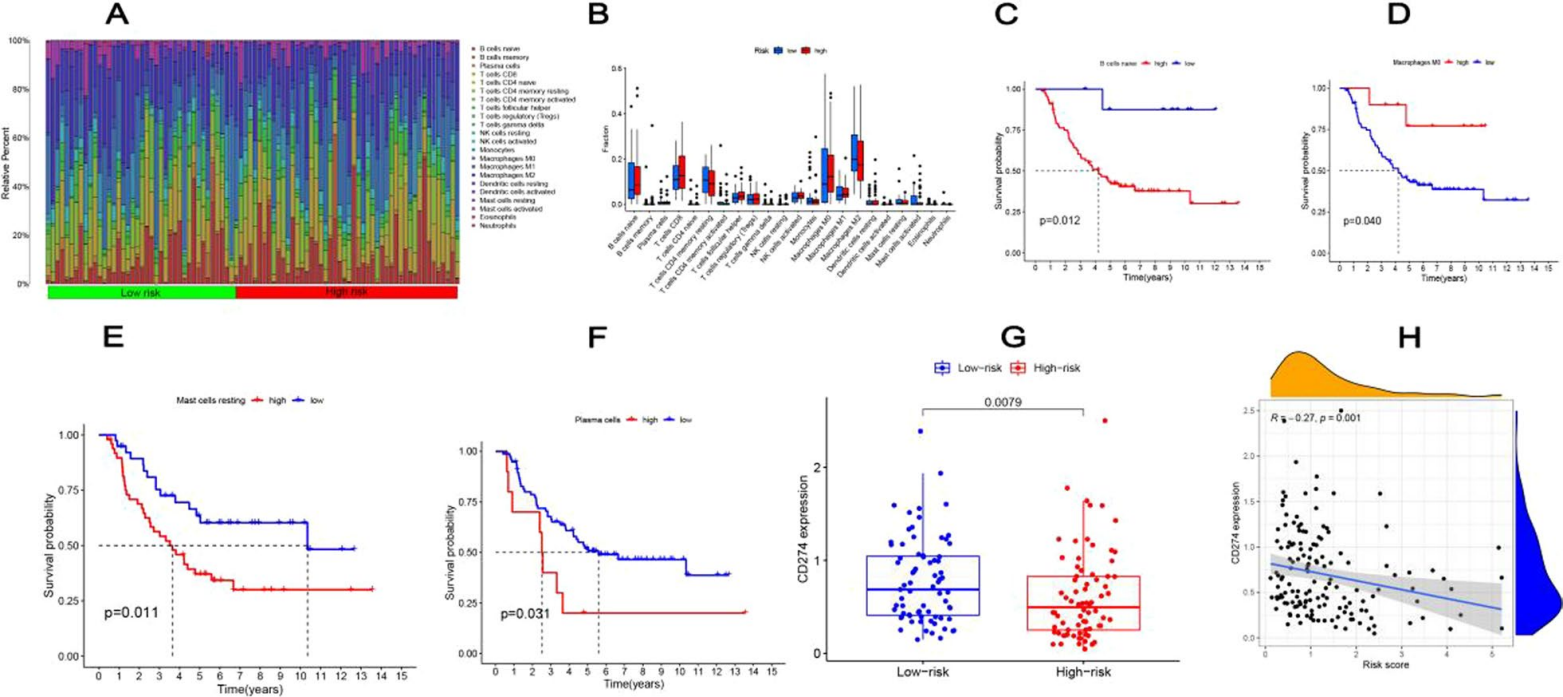
The relationship between the risk model and the infiltration abundances of 22 immune cells. **A** The relative infiltration abundances of the 22 types of immune cells. **B** Comparison of TIICs infiltration abundance between the high- and low-risk groups. Kaplan–Meier survival analysis of **C** B cells naïve, **D** macrophages M0, **E** mast cells resting, and **F** plasma cells for patients with high- and low abundance. **G** Box plot and **H** correlation graph showed the differences in immune checkpoint CD274 in high and low-risk groups

CIBERSORT was used to estimate the 22 kinds of TIICs infiltration abundance (Fig. 6A). 88 patients with p < 0.05 were selected for the subsequent analysis. There were no statistical differences of TIICs infiltration abundances in the two risk groups (Fig. 6B). According to the K-M analysis, the expression of four immune cells, including B cells naïve (cutpoint = 0.007, *p* = 0.012), macrophages M0 (cutpoint = 0.384, *p* = 0.040), mast cells resting (cutpoint = 0.0003, *p* = 0.011), and plasma cells (cutpoint = 0.028, *p* = 0.031) were associated with the prognosis (Fig. 6C-F). The high abundance of Macrophages M0 showed that patients had a good prognosis (*p* < 0.05) while B cells naïve, mast cells resting, and plasma cells showed a poor prognosis. Immune checkpoint gene CD274 showed a negative correlation with the risk score. (R = -0.27, *p* = 0.001) (Fig. 6H), and a higher expression in the low-risk group (Fig. 6G). To explore the prognostic value of the risk score for immune-checkpoint therapy, patients in the IMvigor210 cohort were assigned to high- or low-risk groups. K–M analysis showed that the low-risk group patients had a significantly better prognosis (*P* < 0.001) (Additional file 1: Fig. S4A). We also compared the differences of immunosuppressive benefits between the two risk groups, the low‐risk group patients had a higher complete response (CR)/partial response (PR) rate (Additional file 1: Fig. S4B).

**Fig. 7:**
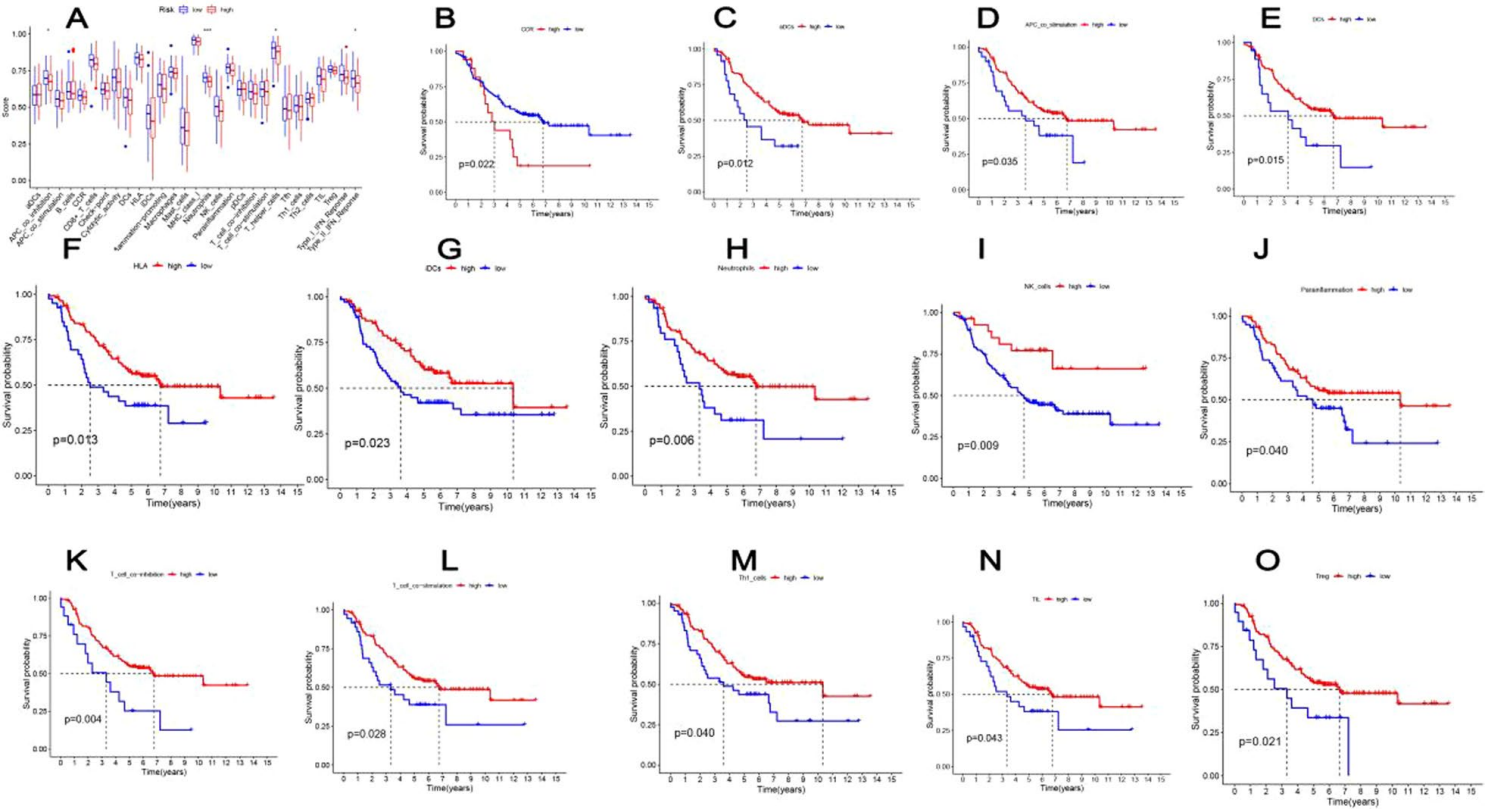
Kaplan–Meier analysis for 29 immune functions. **A** K–M curve to compare the difference of immune functions between the high- and low-risk groups, respectively. **B**–**O** *p < 0.05; **p < 0.01; ***p < 0.001, n. s. statistically insignificant

4 kinds of immune functions, including APC-co-inhibition, neutrophils, T-helper cells and type-II-IFN-reponse, were suppressed in the high-risk group (Fig. 7A). According to the survival analysis, except for the immune function of CCR, the more active of the immune functions of aDCs (cutpoint = 0.486, *p* = 0.012), APC co stimulation (cutpoint = 0.491, *p* = 0.035), DCs (cutpoint = 0.407, *p* = 0.015), HLA (cutpoint = 0.784, *p* = 0.013), neutrophils (cutpoint = 0.646, *p* = 0.006), iDCs (cutpoint = 0.414, *p* = 0.023), NK cells (cutpoint = 0.584, *p* = 0.009), T cell co inhibition (cutpoint = 0.495, *p* = 0.004), parainflammation (cutpoint = 0.743, *p* = 0.040), T cell co stimulation (cutpoint = 0.547, *p* = 0.028), TIL (cutpoint = 0.627, *p* = 0.043), Th1 cells (cutpoint = 0.455, *p* = 0.040) and Treg(cutpoint = 0.721, *p* = 0.021), the better prognosis of NBL patients(Fig. 7B-O).

### The tumor mutational burden of NBL samples

**Fig. 8:**
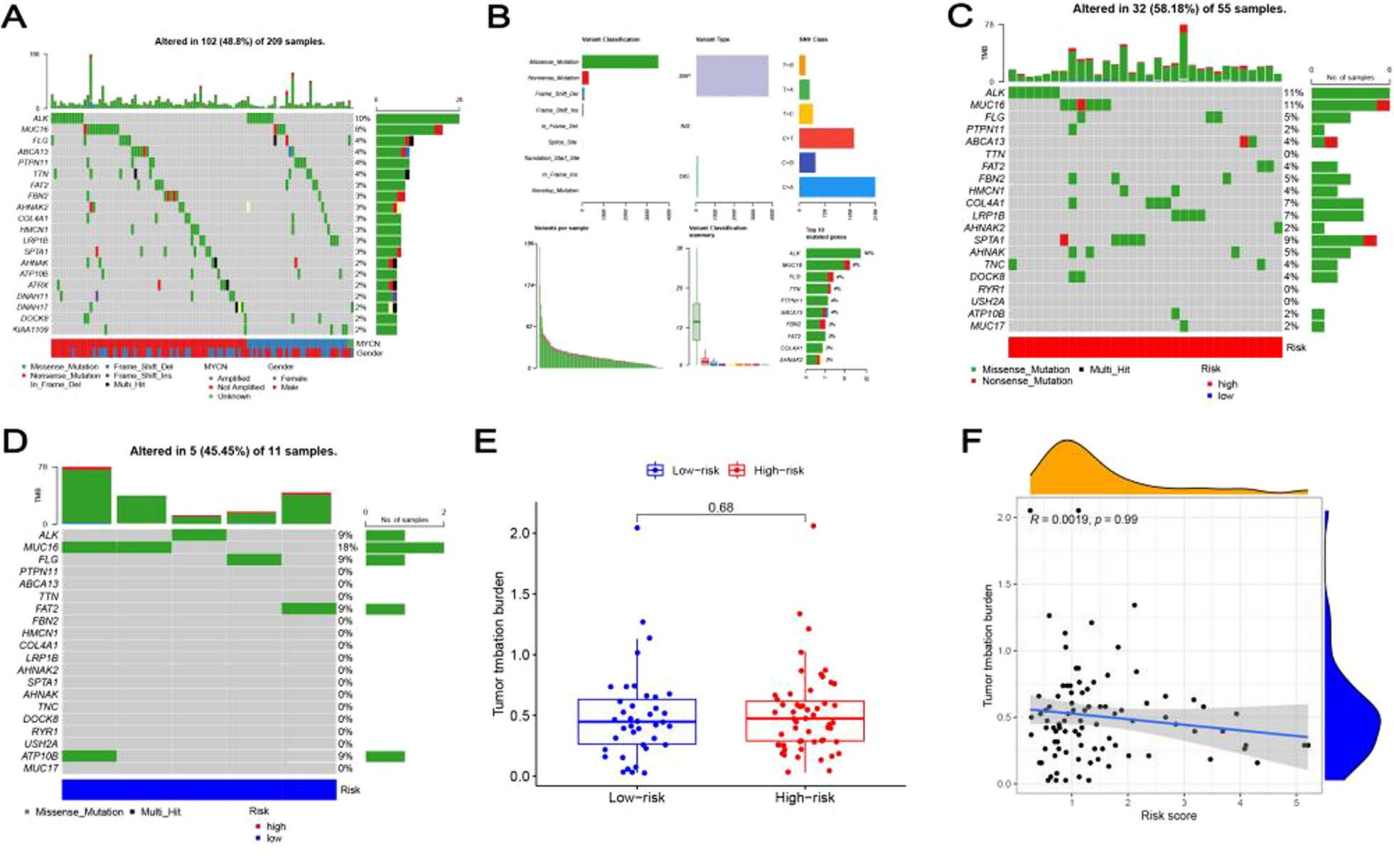
The mutation profile and TMB in the low- and high-risk groups. **A** waterfall plot of the top 20 most mutated genes in different patients shows the relationship with gender and MYCN status. **B** Counting mutations in seven common classifications, separately. **C**–**D** Waterfall plot of top 10 mutated genes in MYCN-related DEIRGs between the two risk groups. **E** Boxplot and **F** correlation graphs indicated the difference in TMB in the two risk score groups

We showed the top 20 most mutated genes in somatic mutation profiles patients. The waterfall plot was used to distinguish different mutation types and show the relationship between gender and MYCN status (Fig. 8A). Missense mutations, C > A mutation, and single-nucleotide polymorphism (SNP) accounted for the majority of different classifications. In addition, counting each sample separately, the maximum mutations were 186. The box plot showed the number of variant classifications in the different samples. The top 3 mutated genes were ALK (10%), MUC16 (8%), FLG (4%) (Fig. 8B). To identify the differences of the TMB, we compared the top 20 most common mutated genes between the two risk groups. ALK, MUC16, FLG, FAT2, and ATP10B were the commonly mutated genes in the low-risk group (Fig. 8C), while ALK, MUC16, FLG were commonly mutated genes in the high-risk group (Fig. 8D). Accordingly, ALK (10%) was the most common mutant gene in the 149 NBL patients. The boxplot and correlation graphs showed that the TMB had no significant difference in the two risk groups (Fig. 8E, F).

## Discussion

NBL is an embryonal malignancy originating from neural crest cells, which is frequently characterized by the amplification and overexpression of MYCN oncogene [13, 14]. MNA is associated with high-risk disease, advanced-stage disease, metastatic behavior, rapid progression, poor survival, and unfavorable prognosis [15, 16]. The MYCN-amplified subtype constitutes the most aggressive and least treatable form of NBL. Despite intensive treatment models, the long-term survival of high-risk NBL is still around 50% [14]. Tumors with MNA have a unique gene expression profile, and the altered gene expression is considered to be the primary oncogenic function of MYCN [17]. However, targeting this oncogene remains challenging in clinical treatment. In this study, we analyzed EDGs between the two MYCN status groups. Based on the ImmPort and InnateDB databases. 343 DEIRGs, including 320 down-regulated and 23 up-regulated genes in the MNA group were extracted from DEGs based on the IRGs list. Functional enrichment analysis showed that these DEIRGs participated in leukocyte adhesion-related signaling pathways, including the regulation of leukocyte cell–cell adhesion, leukocyte cell–cell adhesion, leukocyte mediated immunity, and positive regulation of leukocyte cell–cell adhesion. Several cell adhesion molecules (CAMs) are involved in leukocyte adhesion. These CAMs are associated with the adhesion and metastatic behavior of NBL cells [18, 19]. These enriched pathways might mean that leukocyte adhesion is partially responsible for the malignant progression of NBL.

Based on the WGCNA, we screened 3 gene co-expression modules, of which the turquoise and grey modules were closely correlated with MNA (*p* < 0.01), and a total of 228 DEIRGs were selected for the following research. Through univariate and multivariate regression analyses, we finally selected five genes (PLXNC1, CHGA, PTGER1, SHC3, and TRIM55) to construct the risk model. The survival curves showed a poorer prognosis in the high-risk group which was validated in the GSE49711 and the E-MTAB-8248 datasets. Two kinds of tumor cells immune escape mechanisms have been founded at present. TIDE was used to identify the potential factors of tumor cells immune evasion mechanisms. However, except for dysfunction, the other three TIDE, MSI, and exclusion showed no significant differences in the two risk groups. TIDE was calculated in different cancer databases. The number of our samples is limited and all of them were from the TARGET database, which may lead to bias. Larger research is needed to explore the specific mechanisms in the future.

Here, the prediction accuracy of the risk AUC was better than the TIS and TIDE. Therefore, the signature based on the five MYCN-related DEIRGs had an excellent predictive effect. The signature may be helpful to evaluate the prognosis and update treatment for NBL patients. Heterogeneous diseases have a large number of clinicopathological characteristics and risk factors. So, we should analyze whether the risk model can be an independent factor. Through univariate and multivariate analyses, we concluded that the risk score was an independent prognosis factor in our model. However, former studies have proved that MYCN is related to poor prognosis in NBL patients. Subsequently, we performed univariate and multivariate analyses in the E-MTAB-8248 and GSE49711 datasets, the results showed that the risk score, age, and MYCN all can serve as independent prognostic factors for NBL patients. After our analysis, data collections in the TARGET database can lead to bias in our model. Thus, more datasets are needed to identify the result.

When it comes to clinical characteristics, the risk model was strongly correlated with age, histology, MKI, and COG. International Neuroblastoma Risk Group [20] uses histologic category, age, grade, MYCN status, DNA ploidy, etc., as a risk group for NBL patients, which helps to develop individualized treatment options according to different risk characteristics.

Through univariate Cox regression analysis, we selected 12 genes, including CRABP1, AMH, CHGA, PTGER1, SHC3, GAL, MAPT, PLXNC1, SCG2, LIFR, TRIM55, and OPTN. These 12 genes are associated with the progression and prognosis of cancer. For example, the expression of CRABP1 of cancers from various origins are significantly different. High levels of CRABP1 were detected in NBL but not in NSCLC, ovarian cancer, and glioblastoma. By DNA methylation analysis, CRABP1 was identified as a hypermethylated target gene of ovarian cancer [21]. The decreased expression of CRABP1 is associated with the poor prognosis of serous and clear cell ovarian adenocarcinoma [22]. MAPT is aberrantly expression in some cancers. The aberrantly expression of MAPT is an independent prognostic factor in prostate cancer, and its knockdown can reduce cell growth [23]. Marachelian et al. [24]. first reported that the mRNA quantification of 5-related genes (CHGA, DCX, DDC, PHOX2B, and TH) could be used as biomarkers for relapsed/refractory NBL. In a pan-cancer study, the overall expression of PLXNC1 is up-regulated in primary and metastatic tumors and may be associated with a more aggressive cancer phenotype [25]. SCG2 is an independent prognostic factor in colorectal cancer, which is associated with macrophage polarization and immune infiltration [26]. The expression of gene LIF/LIFR is overexpressed in solid tumors, and plays an essential role in cancer metastasis, progression, and invasion [27]. PTGER1, one of the prostaglandin receptors, conjugates G-proteins to activate the protein kinase C. DNA methylation is a critical link in tumor transformation, PTGER1 is closely related to DNA methylation in non-functioning adrenocortical adenoma [28]. In addition, the high-level expression of PTGER1 is correlated with a poor prognosis in clear cell renal cell carcinoma [29]. SHC3 is ectopically overexpressed in various cancers and associated with their progression. The interaction between SHC3 and HIF- 1α may prevent NBL cells from hypoxia. 8 IRGs, including TRIM55, were associated with the survival and clinical features of lung squamous carcinoma (LUSC) [30]. By augmenting protein degradation of Snail1 via promoting the ubiquitination pathway, TRIM55 inhibits the malignant behavior of lung adenocarcinoma [31]. The expression of OPTN is down-regulated in urothelial carcinoma [32]. Overexpression of OPTN increases mitophagy and is related to worse prognosis in hepatocellular carcinoma (HCC) patients [33].The expression of GAL up-regulated in neuroblastic cancers. The expression of Galanin and galanin receptors are correlated with abnormal differentiation of neuroblastic tumors [34]. However, except for MAPT, the other 11 genes have not been reported to be associated with the prognosis of NBL. MAPT microarray values are associated with MNA. High level expression of MAPT can improve the survival of NBL, which is consistent with the apoptosis-effector and proliferation genes [35]. ADAM22, GAL, KLHL13, and TWLST1 are up-regulated in ultra-high risk NBL, and they are overexpressed in patients with MNA [36]. Furthermore, GAL can be an independent prognosis factor for high-risk NBL. These twelve genes were closely associated with MNA. Some genes have been reported to be associated with cancer progression. So, further researchs are needed to explore the relationship between the 12 genes and MNA and their roles in NBL prognosis.

TMB can be used as a new immunotherapy biomarker. We analyzed the mutation profiles of NBL patients to explore the potential mechanisms. The C > A mutations accounted for the majority. The three commonly mutated genes were ALK (10%), MUC16 (8%), and FLG (4%). MUC16, encoding cancer antigen 125 (CA125), promotes tumor proliferation and metastasis and can play an immunosuppressive role. ALK is the most common single-gene alteration in family NBL. Bresler et al. [37] found that ALK amplification and MNA concomitantly occurred in almost all cases, and both of them indicated that NBL had a poor prognosis. TMB is associated with the overall survival of malignant tumors. By analyzing 459 NBL patients, a higher somatic mutational burden is associated with lower survival in NBL [38]. According to the waterfall plot, there was no significant difference in TMB between the two groups, and there was no significant correlation between TMB and the risk score. However, the top 20 most common mutated genes were different. Overall, NBL patients have low mutational frequencies. Therefore, larger data and specific researches are needed to analyze the relationship between TMB and the prognosis.

By GSEA functional enrichment analysis, the 12 MYCN-related DEIRGs may be involved in the pathogenesis of NBL. The high-risk group was closely associated with DNA replication, cell cycle, mismatch repair, ribosome, and spliceosome. In contrast, the low-risk group was correlated with complement and coagulation cascades, PPAR signaling pathway, drug metabolism of xenobiotics by cytochrome P450, drug metabolism cytochrome P450, retinol metabolism. and cell cycle. DNA replication is a potential pathway for the initiation and treatment of NBL. Studies have shown that MYC proto-oncogene participates in DNA replication through transcriptional or nontranscriptional mechanisms and can control the initiation of DNA replication [39]. Alterations of defects-related genes associated with DNA damage response (DDR) are frequently observed in the high-risk NBL [40]. MYCN is involved in regulating gene expressions related to ribosomal biogenesis. MYC transcription factors induced ribosome hyperactivity is related to the poor prognosis of NBL [41]. By RNA sequencing analysis, the high level expression of spliceosome factors is associated with high-risk NBL [42]. PPAR agonists and target the retinol signaling pathway may be involved in the treatment of NBL [43]. Ethanol Induce Cytochrome P450 protects against MPP + induced NBL toxicity [44]. These studies have proved that these pathways can serve as potential therapeutic targets for NBL.

Growing evidence shows that the efficacy of immunotherapy is associated with TME. The dynamic balance of anti-tumor and pro-tumor immune cells lead to the malignant progression of tumors. The TIICs infiltration may affect immunotherapy. Several studies have analyzed the relationship between TIICs infiltration abundance and MNA [45]. Therefore, we explored the immune signatures of NBL. We used ssGSEA, CIBERSORT, and immune checkpoint to analyze the TME. The TIICs infiltration showed no significant differences in the two risk groups. According to the survival curves, four TIICs infiltration abundance, including the B cells naïve, macrophages M0, mast cells resting, and plasma cells were screened associated with NBL prognosis. The high abundance of B cells naïve, plasma cells, and mast cells resting were tightly related to a poor prognosis. APC-co-inhibition, neutrophils, T-helper cells and type-II-IFN-reponse were suppressed in the high-risk group. Except for CCR, the other 13 showed a better prognosis in a more active of immune functions. Several kinds of studies are similar to the conclusion of this study [46, 47]. However, some reports are different [48]. Therefore, we still require further analysis to realize the relationship between immune functions and the prognosis, which may be helpful for immunotherapy. The high level expression of the immune checkpoint is related to immune cell infiltration and poor prognosis of children with solid tumors. Immunocheckpoint inhibitor is a novel method of immunotherapy. In this study, the immune checkpoint gene CD274 was negatively correlated with the risk score, and the level expression of CD274 was higher in the low-risk group. The conclusion is consistent with other studies [49]. The result suggests that the low-risk group has a better therapeutic effect on anti-PD-L1 therapy. Some reports have indicated that anti-PD-L1 immunotherapy can enhance the response of T-cell to control tumor progression [50]. According to the IMvigor210 cohort, we concluded that patients in the low-risk group had a higher CR/PR rate. Thus, this immune checkpoint can be considered as a predictive marker and a potential therapeutic target.

## Conclusion

Our study has several limitations. First, although we used the GEO database for external verification, it is still a retrospective study, lacking long-term follow-up. Second, the role of these five genes in NBL progression and prognosis should be confirmed using in vivo and vitro experiments. Therefore, we still need corresponding clinical trials. In conclusion, we selected and analyzed DEIRGs between the two MYCN status and then constructed a well-performed five immune-related gene signature to predict the prognosis of NBL patients. A risk AUC indicated an excellent prediction result combined with the independent prognostic factor. GSEA indicated the potential pathways of NBL, which may help identify the underlying mechanisms. Furthermore, we explored the value of immune signatures and immune checkpoints in different risk score groups, which will help identify the prognostic indicator and immune treatment target. Collectively, these results provide new treatment strategies for NBL patients.

## Supplementary Information

**Additional file 1**. **Fig. S1.** The correlation between the prognostic genes and overall survival. **Fig. S2.** Identification of the independent prognosis factor in the risk model. **Fig. S3.** Identification of the independent prognosis factor in the GSE49711 dataset. **Fig. S4.** Immune signature predicts immunotherapy benefit.

## Data Availability

Our data can be found in the Therapeutically Applicable Research to Generate Effective Treatments (TARGET) database (www.ocg.cancer.gov/programs/target). The Gene Expression Omnibus (GEO, www.ncbi.nlm.nih.gov/geo/, GSE49711) database. The ArrayExpress (www.ebi.ac.uk/arrayexpress, E-MTAB-8248) database. The IMvigor210 cohort (http://research-pub.gene.com/IMvigor210CoreBiologies). The ImmPort database (www.immport.org/home) and the InnateDB database (www.innatedb.ca).

## Abbreviations

AHSCT: Autologous hematopoietic stem cell transplantation
CA125: Cancer antigen125
CAMs: Cell adhesion molecules
CR: Complete response
DDR: DNA damage response
DEGs: Differentially expressed genes
DEIRGs: Differentially expressed immune-related genes
FDR: False discovery rate
GEO: Gene Expression Omnibus
GO: Gene Ontology
GSEA: Gene set enrichment analysis
HCC: Hepatocellular carcinoma
IRGs: Immune-related genes
KEGG: Kyoto Encyclopedia of Genes and Genomes
K-M: Kaplan–Meier
LUSC: Lung squamous Carcinoma
MNA: MYCN amplification
NBL: Neuroblastoma
NCCs: Neural crest cells
OS: Overall survival
PR: Partial response
ROC: Receiver operating characteristic
ssGSEA: Single-sample gene set enrichment analysis
TARGET: Therapeutically Applicable Research to Generate Effective Treatments
TIDE: Tumor Immune Dysfunction and Exclusion
TIICs: Tumor-infiltrating immune cells
TIS: Tumor inflammation signature
TMB: Tumor mutation burden
TME: Tumor microenvironment
WGCNA: Weighted gene coexpression network analysis
