# Supplementary material for: A signature based on five immune-related genes to predict the survival and immune characteristics of neuroblastoma
Source: BMC Med Genomics. 2022 Nov 23;15:242. doi: 10.1186/s12920-022-01400-y (PMC9685875; doi:10.1186/s12920-022-01400-y)
Supplement: Supplementary file 1 — Additional file 1. Fig. S1. The correlation between the prognostic genes and overall survival. Fig. S2. Identification of the independent prognosis factor in the risk model. Fig. S3. Identification of the independent prognosis factor in the GSE49711 dataset. Fig. S4. Immune signature predicts immunotherapy benefit. [file 12920_2022_1400_MOESM1_ESM.docx]

**
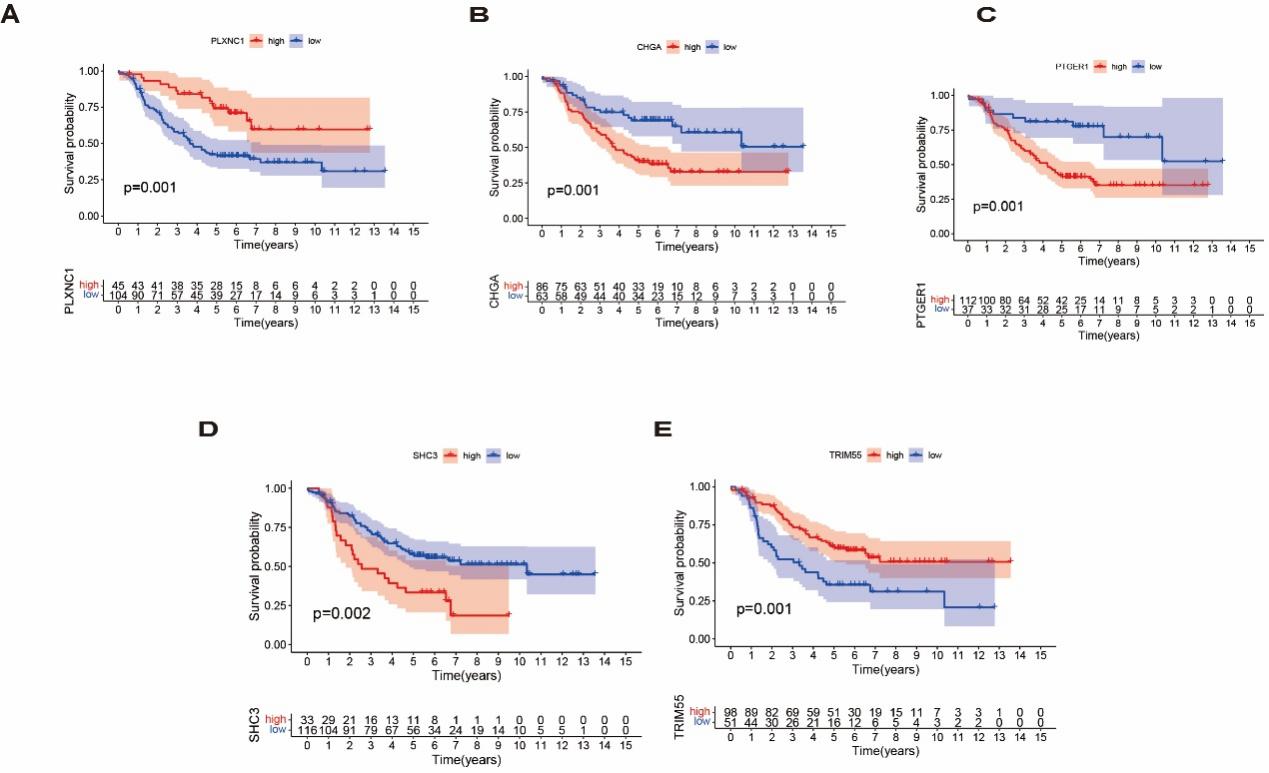
**

**Additional Fig. S1** The correlation between the prognostic genes and overall survival. The Kaplan-Meier analysis of (**A**) PLXNC1, (**B**) CHGA, (**C**) PTGER1, (**D**) SHC3, (**E**) TRIM55 in the high- or low-risk groups


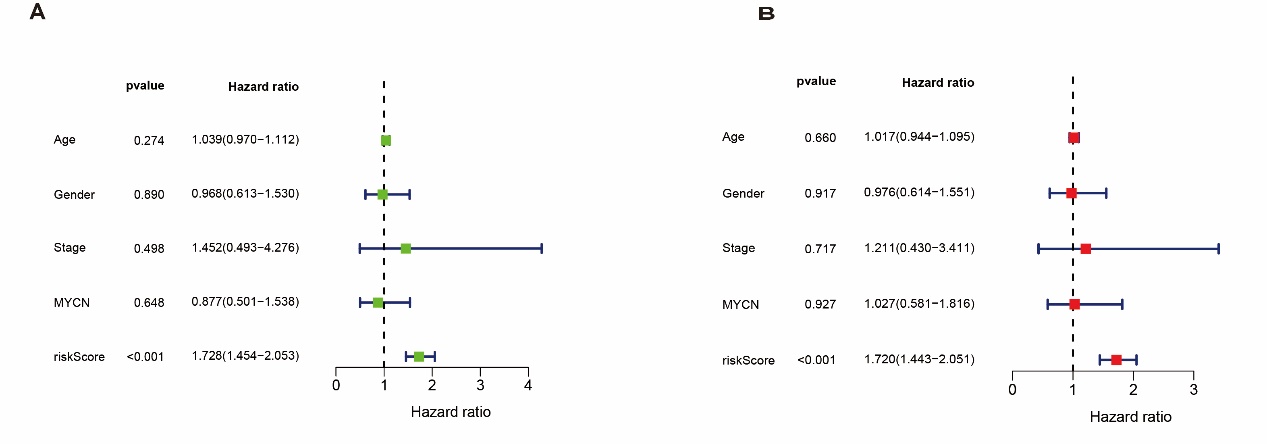


**Additional Fig. S2** Identification of the independent prognosis factor in the risk model. The independence of the risk model was assessed by (**A**) univariate and (**B**) multivariate Cox regression analysis.

**
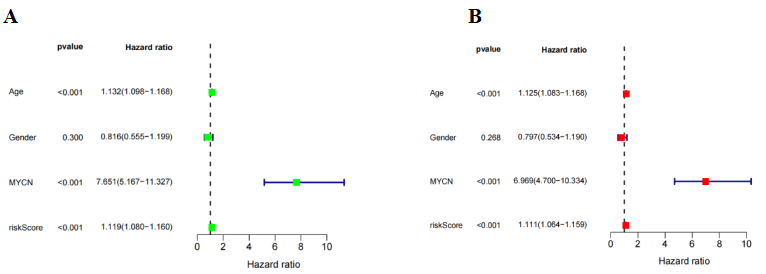
**

**Additional Fig. S3** Identification of the independent prognosis factor in the GSE49711 dataset. The independence of the risk model was assessed by (**A**) univariate and (**B**) multivariate Cox regression analysis.


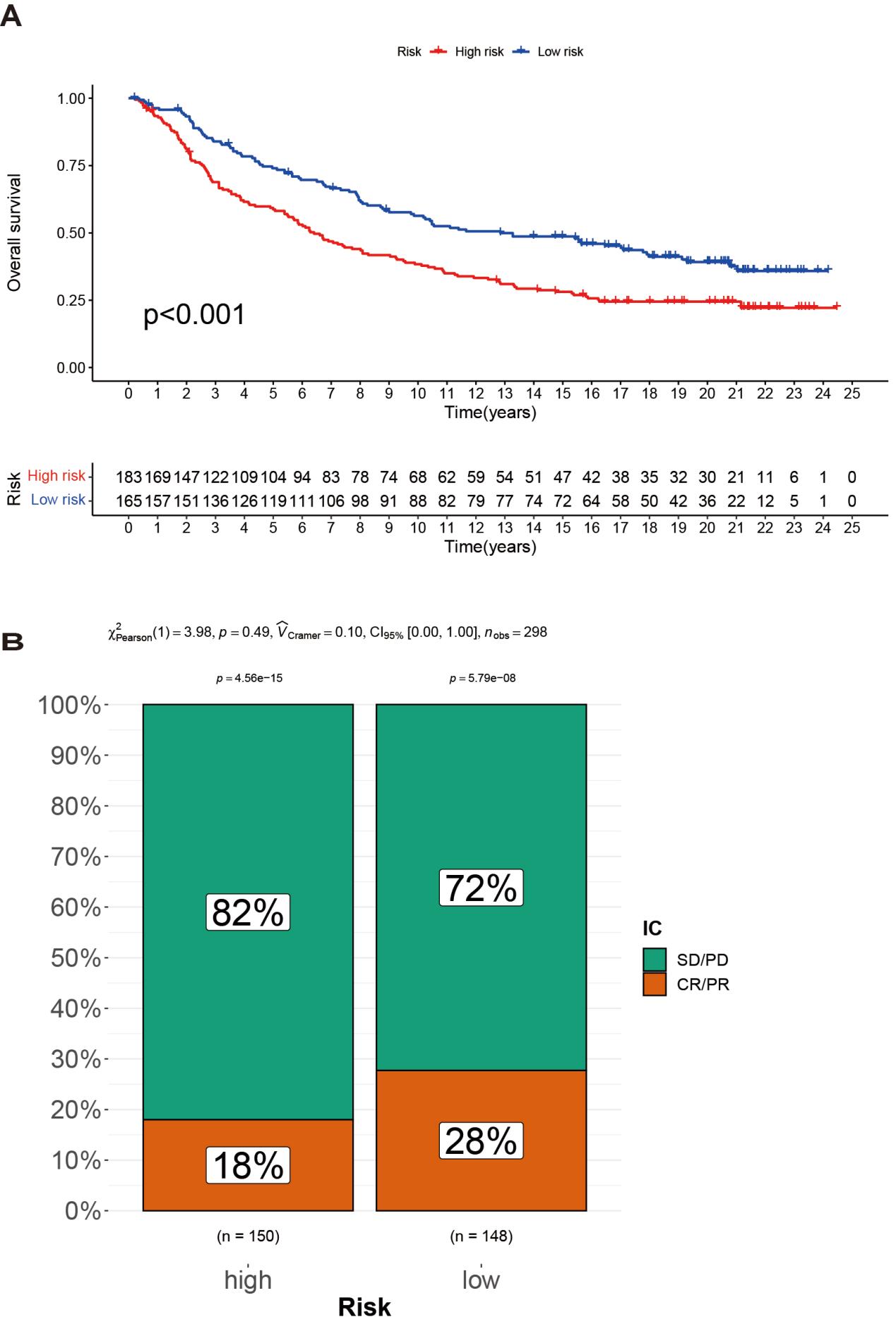


**Additional Fig. S4** Immune signature predicts immunotherapy benefit. (**A**) The Kaplan-Meier analysis for overall survival (OS) of patients based on the risk stratification in the IMvigor210 cohort. (**B**) Rate of clinical response (CR/PR and SD/PD) to immunotherapy in high- or low-risk groups.
